# Supplementary material for: No carbon storage in growth-limited trees in a semi-arid woodland
Source: Nat Commun. 2023 Apr 7;14:1959. doi: 10.1038/s41467-023-37577-8 (PMC10081995; doi:10.1038/s41467-023-37577-8)
Supplement: Supplementary file 1 — Supplementary Information [file 41467_2023_37577_MOESM1_ESM.pdf]

Supplementary Info

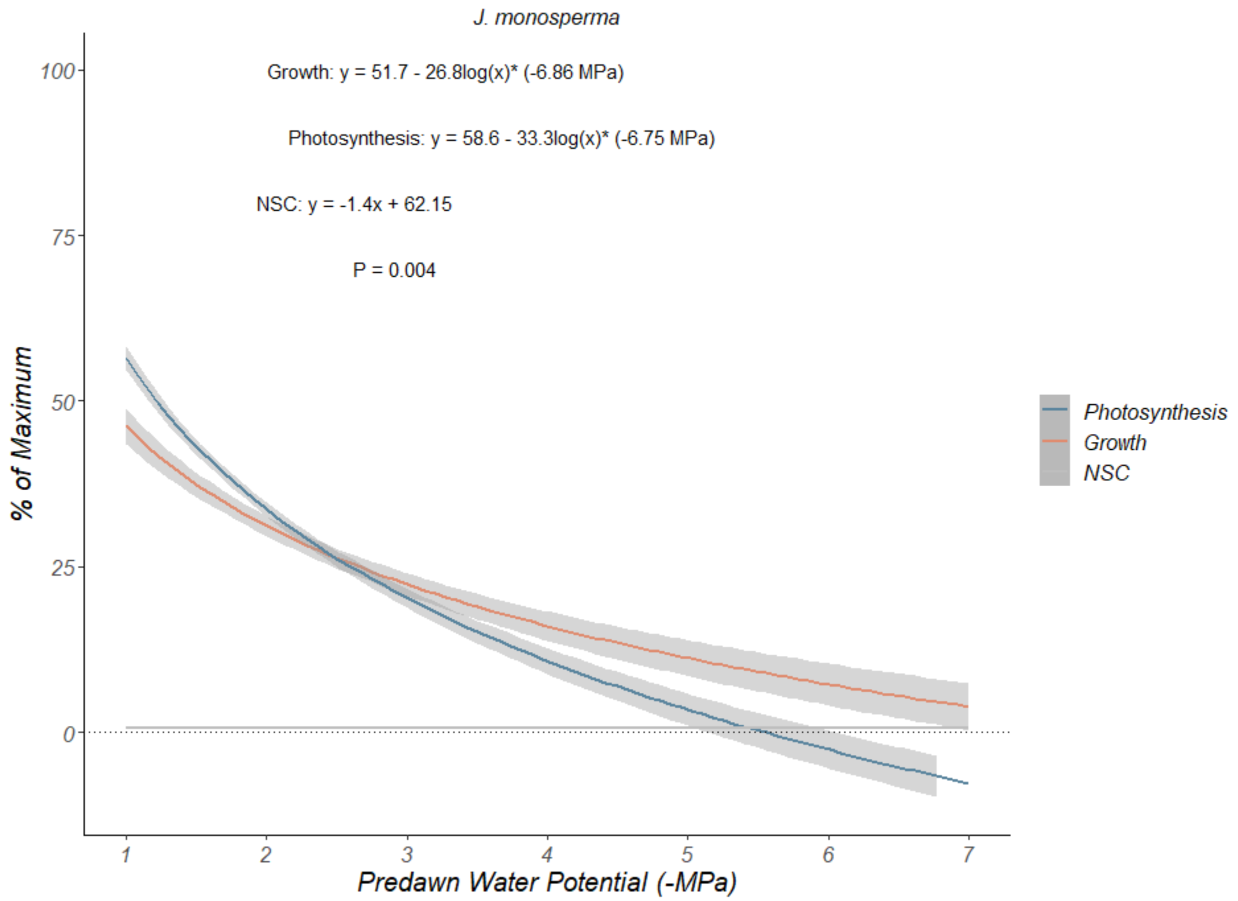

Supplementary Figure 1. Regression of growth, photosynthesis, and June starch concentrations for the needle, twig, bole, and root, against ψ_{pd} for *J. monosperma*. Lines reflect point estimates of the regression coefficient for growth, photosynthes, & starch, separately. Grey areas around lines indicate 95% confidence intervals. A separate analysis of NSC for needle and twig only across the entire growing season can be viewed in Fig. 1B. P-values in each figure correspond to the results of an ANOVA for a comparison between the line of best fit for growth and photosynthesis, respectively.

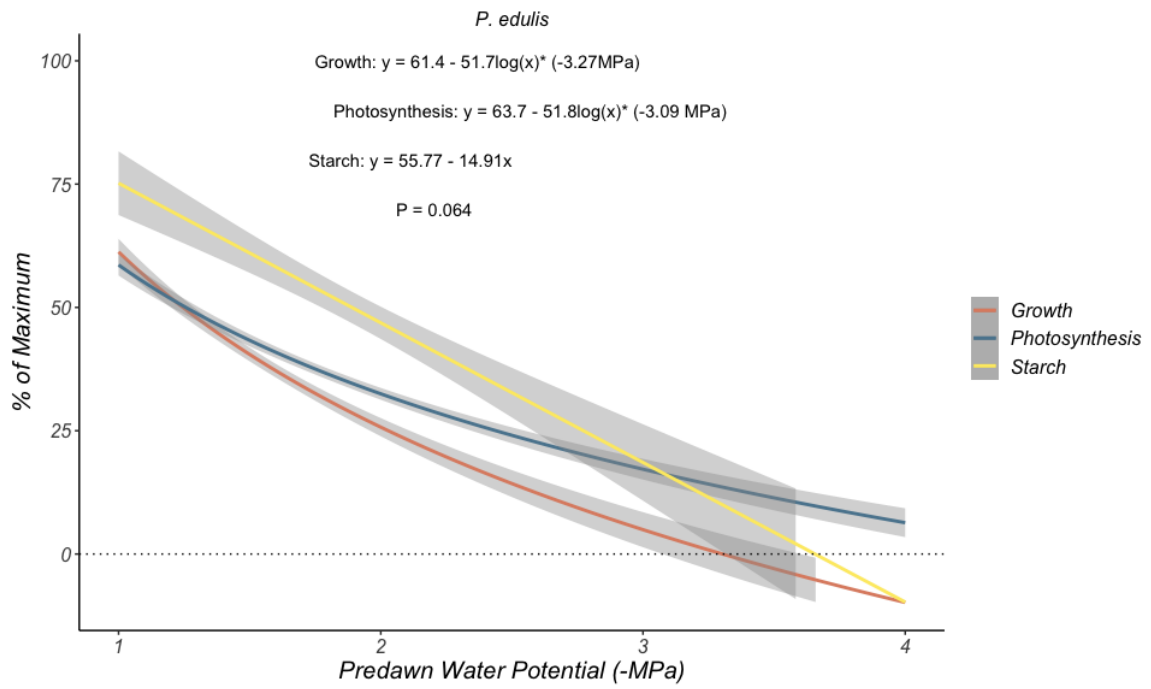

Supplementary Figure 2. Regression of growth, photosynthesis, and June starch concentrations for the needle, twig, bole, and root, against ψ_{pd} for *P. edulis*. Lines reflect point estimates of the regression coefficient for growth, photosynthes, & starch, separately. Grey areas around lines indicate 95% confidence intervals. A separate analysis of NSC for needle and twig only across the entire growing season can be viewed in Fig. 1C. P-values in each figure correspond to the results of an ANOVA for a comparison between the line of best fit for growth and photosynthesis, respectively.

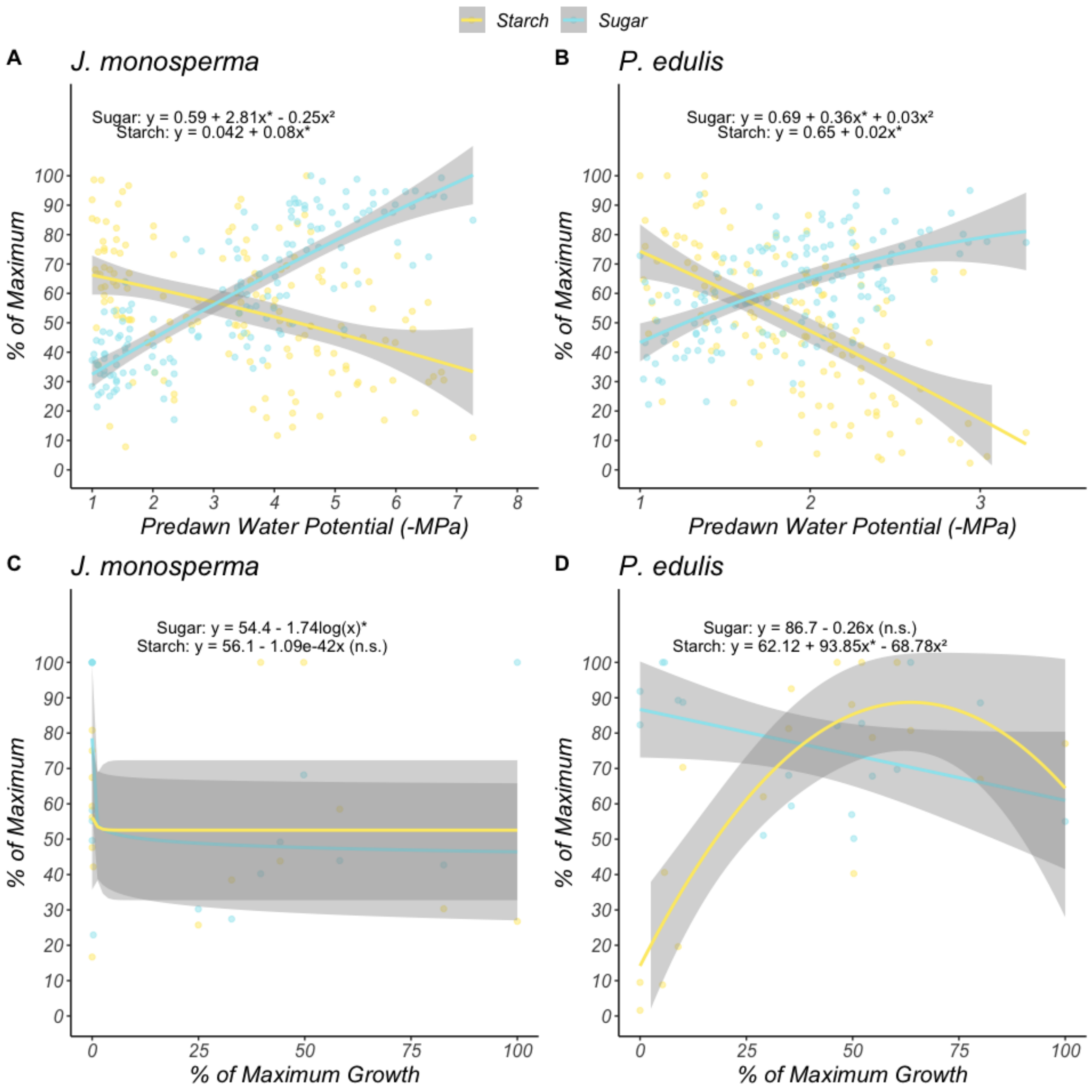

Supplementary Figure 3. Results of the regression of whole-tree average (needle, branch, bole, and root) sugar and starch (June only) against ψ_{pd} and % of maximum growth. Lines reflect point estimates of the regression coefficient for sugar & starch against predawn water potential (A & B) and growth (C & D). separately. Grey areas around lines indicate 95% confidence intervals. A separate analysis of NSC for needle and twig only across the entire growing season can be viewed in Fig. 3.

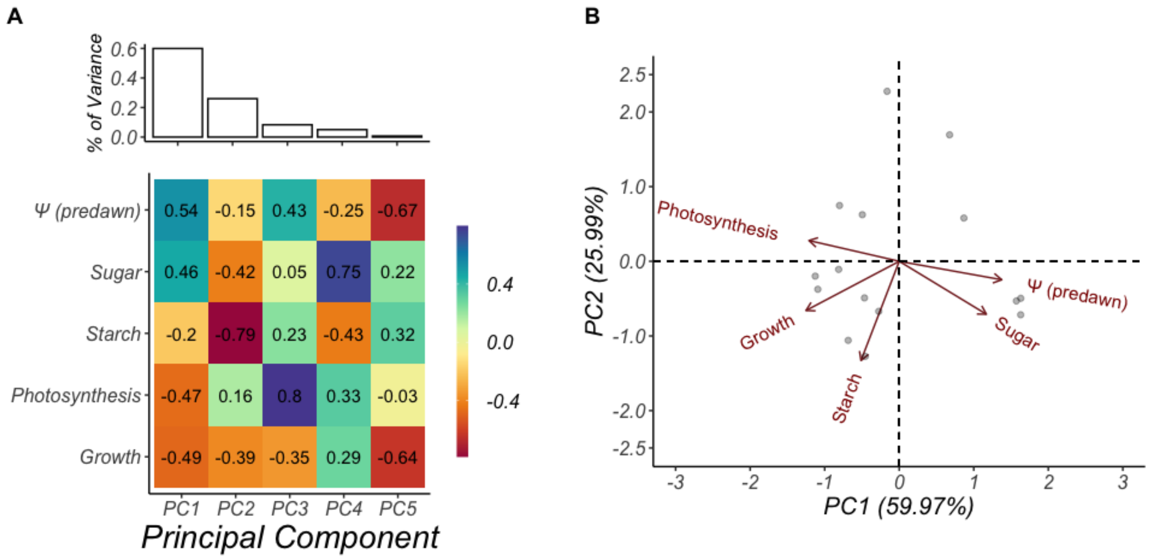

Supplementary Figure 4. Results of the principal components analysis on carbon supply and demand variables including bole, root, twig, and needle NSC for June only. PC1 explained 59.9% of the total variance in the data, and together PC1 and PC2 explain 85% of the total variance in the data. As in Figure 4, growth and photosynthesis respond negatively to declining ψ_{pd} along PC1. The orthogonal relationship between growth and photosynthesis observed in Figure 4 vanishes. We conclude this is likely due to the fact that all observation in this analysis are for a single month only, when growth and photosynthesis are highly correlated (see Figure 2B). A separate analysis across the entire growing season (NSC includes only needle and twig) can be viewed in Fig. 4.

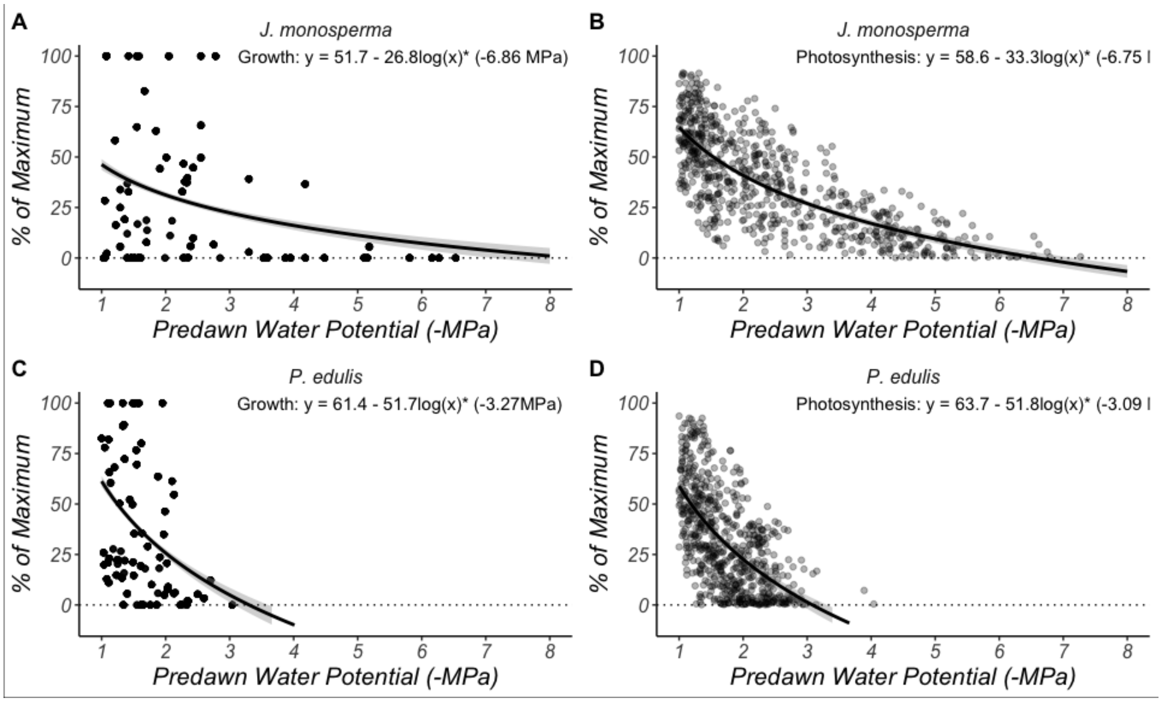

Supplementary Figure 5. Results of the linear regression of % of maximum growth and photosynthesis against ψ_{pd}. For each individual tree, the maximum rate of growth and photosynthesis were identified and all subsequent measurements were expressed relative to that maximum. Points represent individual tree observations for each variable, while linear models (lines) were performed at the species level. Lines represent point estimates of regression coefficients for growth (A & C) and photosynthesis (B & D). respectively. Grey areas around lines indicate 95% confidence intervals.

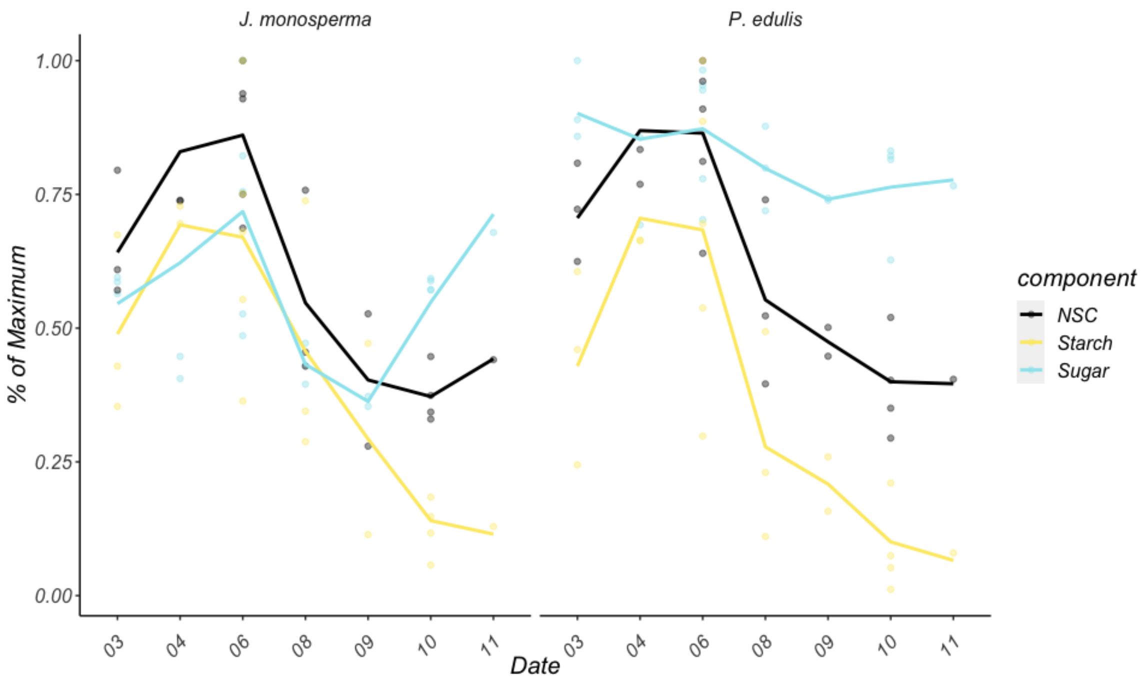

Supplementary Figure 6. Average non-structural carbohydrate concentrations as a function of time. NSC, Sugar, and Starch concentrations were taken as the average species-level value for a given month, among all years and individuals.

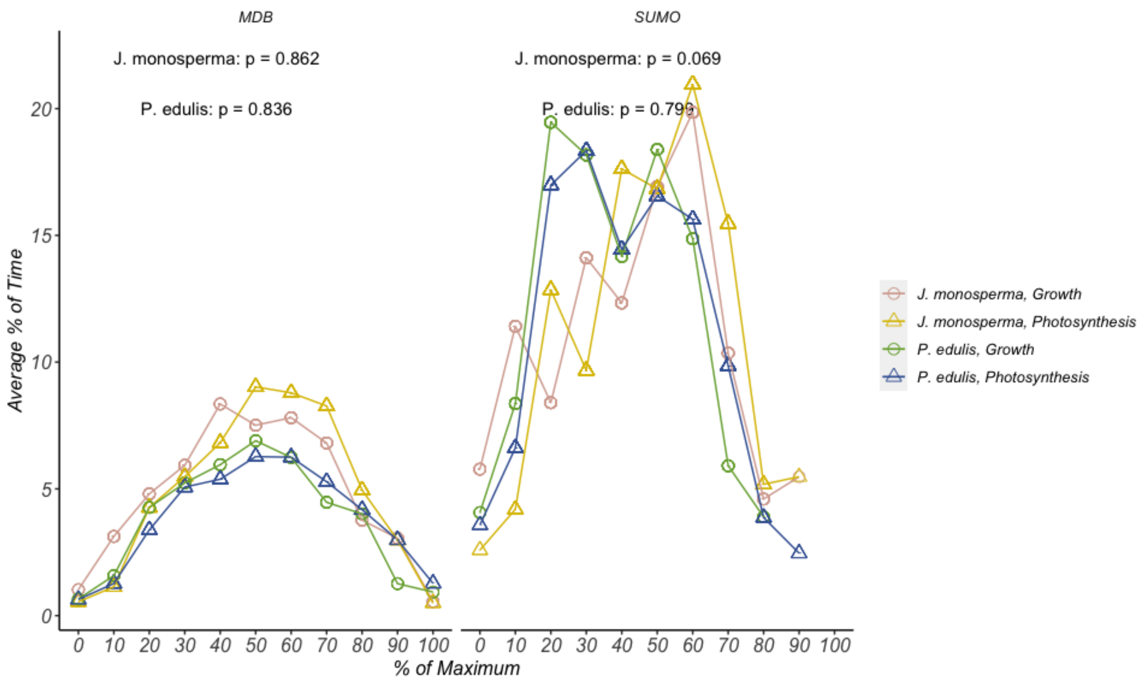

Supplementary Figure 7. When growth was limited, photosynthesis was also limited in both species at SUMO and MDB, though complete cessation was rare. Histograms showing the average % of time at different intervals of growth and photosynthesis (1992 - 2016 at MDB; 2011 - 2017 at SUMO) are shown. Points represent the average frequency of sink (circles) and source (triangles) limitation across all trees within each species, at each site. The % of maximum for both parameters was estimated using interpolations from the linear-log model fit to the original % of maximum data. We used a two-sided Kolmogorov-Smirnov test to evaluate the distribution of each parameter for each species and test for significant differences, which were not found (Supplementary Table 3).

**Species**	**Variable**	**r_squared**	**p-value**	**Comparison**
P. edulis	Growth	0.0650	<0.05	% Maximum ~ Predawn water potential
J. monosperma	Growth	0.1086	<0.05	% Maximum ~ Predawn water potential
P. edulis	Photosynthesis	0.3770	<0.05	% Maximum ~ Predawn water potential
J. monosperma	Photosynthesis	0.4740	<0.05	% Maximum ~ Predawn water potential

Supplementary Table 1. Results of the linear-log model, comparing the response of % maximum growth and photosynthesis to changes in ψ_{pd}.

Estimate	Std..Error	t.value	P	Variable	Species
51.69544	1.1593856	44.58865	0	Growth	*J. monosperma*
-26.84210	1.2774878	-21.01163	0	Growth	*J. monosperma*
61.41258	1.1789959	52.08889	0	Growth	*P. edulis*
-51.70862	2.2906687	-22.57359	0	Growth	*P. edulis*
63.67972	0.8679029	73.37195	0	Photosynthesis	*J. monosperma*
-33.33316	0.9631251	-34.60937	0	Photosynthesis	*J. monosperma*
58.57976	1.1452326	51.15097	0	Photosynthesis	*P. edulis*
-51.79140	2.0249218	-25.57699	0	Photosynthesis	*P. edulis*

Supplementary Table 2. Estimates, standard errors and relevant statistics from the linear-log regression of growth and photosynthesis with water potential shown in Supplementary Table 1.

**Terms**	**Estimate**	**Std. Error**	**t value**	**P**	**Adjusted R-Squared**	**Species**
Intercept	60.651333	0.9230556	65.707125	0	0.2323880	*P. edulis*
Growth:Water Potential	-50.499140	1.8994328	-26.586431	0	0.2323880	*P. edulis*
Growth:Water Potential:Photosynthesis	-4.203251	2.2665808	-1.854446	0.064	0.2323880	*P. edulis*
Intercept	55.066277	0.8779516	62.721315	0	0.2366977	*J. monosperma*
Growth:Water Potential	-29.761904	1.0371800	-28.695022	0	0.2366977	*J. monosperma*
Growth:Water Potential:Photosynthesis	3.836569	1.3583893	2.824351	0.004	0.2366977	*J. monosperma*

Supplementary Table 3. Results of the two-sided ANOVA comparing the linear-log regression of growth and photosynthesis within and between species. Results indicate no significant difference between variables, within species, but within variable comparisons between species were significantly different.

D	P.value	Species	Site
0.12982891	0.06852807	*J. monosperma*	SUMO
0.06572694	0.79874763	*P. edulis*	SUMO
0.07960275	0.86183870	*J. monosperma*	MDB
0.10030759	0.83623870	*P. edulis*	MDB

Supplementary Table 4. Results of the Kolmogorov-Smirnov test evaluating the distribution of growth and photosynthesis within species and sites. Here, the null hypothesis is that the distribution of the variables (growth and photosynthesis) is equivalent. A p-value less than $\alpha = 0.05$ indicates the distribution is not equal. We show that in all cases, photosynthesis and growth occurred at a given % of maximum with equal frequency. Theory would suggest that if growth were more sensitive than photosynthesis to water limitation, these distributions would not be equal.

rowname	PC1	PC2	PC3	PC4	PC5
Standard deviation	1.535413	1.17431	0.8505164	0.6348326	0.3702892
Proportion of Variance	0.471500	0.27580	0.1446800	0.0806000	0.0274200
Cumulative Proportion	0.471500	0.74730	0.8919700	0.9725800	1.0000000

Supplementary Table 5. Proportion of the variance and cumulative proportion of the variance explained by each principal component.

rowname	PC1	PC2	PC3	PC4	PC5
Standard deviation	1.731667	1.139973	0.6447677	0.5016504	0.185512
Proportion of Variance	0.599730	0.259910	0.0831500	0.0503300	0.006880
Cumulative Proportion	0.599730	0.859640	0.9427900	0.9931200	1.000000

Supplementary Table 6. Proportion of the variance and cumulative proportion of the variance explained by each principal component for June data only.

Species	component	r_squared	p-value
J. monosperma	Starch	0.008327269	0.61939323872
P. edulis	Starch	0.441752703	0.00001810008
J. monosperma	Sugar	0.016523295	0.48321832783
P. edulis	Sugar	0.015015845	0.48994098899

Supplementary Table 7. Results of the regression between % of maximum growth against annual canopy (twig and needle) sugar and starch, respectively.

Estimate	Std..Error	t.value	P	Component	Species
32.50418623	5.14433233	6.3184460	0.000	Starch	*J. monosperma*
0.07698262	0.15337866	0.5019122	0.619	Starch	*J. monosperma*
14.06560346	6.12998301	2.2945583	0.028	Starch	*P. edulis*
0.83103954	0.16514689	5.0321234	0.000	Starch	*P. edulis*
54.72343962	5.07585807	10.7811209	0.000	Sugar	*J. monosperma*
-0.10744156	0.15133709	-0.7099486	0.483	Sugar	*J. monosperma*
68.14719253	2.93904621	23.1868394	0.000	Sugar	*P. edulis*
-0.05530352	0.07918037	-0.6984499	0.490	Sugar	*P. edulis*

Supplementary Table 8. Estimates, standard errors, and relevant statistics for the regression between % of maximum growth against annual canopy (twig and needle) sugar and starch, respectively.

Species	component	r_squared	p-value
J. monosperma	Starch	0.02841528	0.532582005
P. edulis	Starch	0.45810649	0.002035673
J. monosperma	Sugar	0.01579073	0.642850622
P. edulis	Sugar	0.18517439	0.074658972

Supplementary Table 9. Results of the regression between % of maximum growth against June whole-tree sugar and starch, respectively.

Estimate	Std..Error	t.value	P	Component	Species
57.8224630	8.4840742	6.8154122	0.000	Starch	*J. monosperma*
-0.1299297	0.2030527	-0.6398817	0.533	Starch	*J. monosperma*
32.1958788	10.1178622	3.1820831	0.006	Starch	*P. edulis*
0.7840949	0.2131976	3.6777842	0.002	Starch	*P. edulis*
64.7274655	9.7352553	6.6487692	0.000	Sugar	*J. monosperma*
-0.1104264	0.2329977	-0.4739378	0.643	Sugar	*J. monosperma*
86.6929318	6.3996986	13.5464086	0.000	Sugar	*P. edulis*
-0.2571409	0.1348507	-1.9068570	0.075	Sugar	*P. edulis*

Supplementary Table 10. Estimates, standard errors, and relevant statistics for the regressions between % of maximum growth and June whole-tree sugar and starch, respectively.

Species	Variable	Model	dAIC	df	weight
P. edulis	Starch	Linear	0.0000000	3	0.642
P. edulis	Starch	Polynomial	1.3342477	4	0.330
P. edulis	Starch	Linear-Log	6.2362367	3	0.028
P. edulis	Starch	Negative Exponential	28.8302422	3	0.000
J. monosperma	Starch	Linear-Log	0.0000000	3	0.689
J. monosperma	Starch	Linear	1.6804705	4	0.298
J. monosperma	Starch	Polynomial	7.9437613	3	0.013
J. monosperma	Starch	Negative Exponential	34.1014985	3	0.000
P. edulis	Sugar	Linear	0.0000000	3	0.397
P. edulis	Sugar	Linear-Log	0.2993318	3	0.342
P. edulis	Sugar	Polynomial	1.3863829	4	0.198
P. edulis	Sugar	Negative Exponential	3.6920637	3	0.063
J. monosperma	Sugar	Polynomial	0.0000000	4	0.947
J. monosperma	Sugar	Linear	5.7871630	3	0.052
J. monosperma	Sugar	Linear-Log	19.9579579	3	0.000
J. monosperma	Sugar	Negative Exponential	292.2169404	3	0.000

Supplementary Table 11. Results of the AIC test to find the best-fitting model when regressing annual canopy NSC and it’s components against predawn water potential. Models with the lowest dAIC within each species and variable were used in the analysis.

Species	Variable	Model	dAIC	df	weight
P. edulis	Starch	Linear	0.000000	3	0.639
P. edulis	Starch	Polynomial	1.926038	4	0.244
P. edulis	Starch	Linear-Log	3.398944	3	0.117
P. edulis	Starch	Negative Exponential	25.714704	3	0.000
J. monosperma	Starch	Linear-Log	0.000000	3	0.445
J. monosperma	Starch	Linear	0.206834	3	0.401
J. monosperma	Starch	Polynomial	2.122656	4	0.154
J. monosperma	Starch	Negative Exponential	22.437969	3	0.000
P. edulis	Sugar	Polynomial	0.000000	4	0.867
P. edulis	Sugar	Linear-Log	3.852474	3	0.126
P. edulis	Sugar	Linear	9.664939	3	0.007
P. edulis	Sugar	Negative Exponential	39.861701	3	0.000
J. monosperma	Sugar	Polynomial	0.000000	4	0.727
J. monosperma	Sugar	Linear	1.959584	3	0.273
J. monosperma	Sugar	Linear-Log	15.681004	3	0.000
J. monosperma	Sugar	Negative Exponential	164.584560	3	0.000

Supplementary Table 12. Results of the AIC test to find the best-fitting model when regressing June whole-tree NSC and it’s components against predawn water potential. Models with the lowest dAIC within each species and variable were used in the analysis.

Species	Variable	Model	dAIC	df	weight
P. edulis	Starch	Polynomial	0.0000000	4	0.648
P. edulis	Starch	Linear-Log	1.2270822	3	0.351
P. edulis	Starch	Linear	13.0823125	3	0.001
P. edulis	Starch	Negative Exponential	20.4546037	3	0.000
J. monosperma	Starch	Linear	0.0000000	3	0.314
J. monosperma	Starch	Negative Exponential	0.1895802	3	0.286
J. monosperma	Starch	Linear-Log	0.2462488	3	0.278
J. monosperma	Starch	Polynomial	1.8892676	4	0.122
P. edulis	Sugar	Polynomial	0.0000000	3	0.863
P. edulis	Sugar	Negative Exponential	5.4476360	3	0.057
P. edulis	Sugar	Linear-Log	5.9424861	4	0.044
P. edulis	Sugar	Linear	6.3481693	3	0.036
J. monosperma	Sugar	Linear-Log	0.0000000	3	0.840
J. monosperma	Sugar	Polynomial	4.7500331	4	0.078
J. monosperma	Sugar	Linear	5.9953132	3	0.042
J. monosperma	Sugar	Negative Exponential	6.0911688	3	0.040

Supplementary Table 13. Results of the AIC test to find the best-fitting model when regressing annual canopy NSC and it's components against predawn water potential. Models with the lowest dAIC within each species and variable were used in the analysis.

Species	Variable	Model	dAIC	df	weight
P. edulis	Starch	Polynomial	0.0000000	4	0.972
P. edulis	Starch	Linear-Log	8.1578568	3	0.016
P. edulis	Starch	Linear	8.8940384	3	0.011
P. edulis	Starch	Negative Exponential	19.7004684	3	0.000
J. monosperma	Starch	Negative Exponential	0.0000000	3	0.362
J. monosperma	Starch	Linear	0.9413686	3	0.226
J. monosperma	Starch	Polynomial	1.0481310	4	0.215
J. monosperma	Starch	Linear-Log	1.2214999	3	0.197
P. edulis	Sugar	Linear	0.0000000	3	0.395
P. edulis	Sugar	Polynomial	0.5056575	4	0.306
P. edulis	Sugar	Negative Exponential	1.8118884	3	0.159
P. edulis	Sugar	Linear-Log	2.0815524	3	0.139
J. monosperma	Sugar	Linear-Log	0.0000000	3	0.522
J. monosperma	Sugar	Polynomial	0.8752701	4	0.337
J. monosperma	Sugar	Negative Exponential	3.2699539	3	0.102
J. monosperma	Sugar	Linear	5.1431773	3	0.040

Supplementary Table 14. Results of the AIC test to find the best-fitting model when regressing annual canopy NSC and it’s components against predawn water potential. Models with the lowest dAIC within each species and variable were used in the analysis.

Model	AIC	dAIC	df	weight	Species
Log	23,259.75	0.000000	3	0.576982645158927	*P. edulis*
Polynomial	23,261.49	1.734757	3	0.242362318642399	*P. edulis*
Negative Exponential	23,262.08	2.331858	4	0.179806802741456	*P. edulis*
Linear	23,272.80	13.044823	3	0.000848233457217	*P. edulis*
Log	21,926.07	0.000000	3	0.838111056337137	*J. monosperma*
Negative Exponential	21,929.40	3.328821	4	0.158656266166689	*J. monosperma*
Polynomial	21,937.19	11.115682	3	0.003232674002724	*J. monosperma*
Linear	21,964.67	38.591543	3	0.000000003493449	*J. monosperma*

Supplementary Table 15. Results of the AIC test to find the best-fitting model when regressing % of maximum growth against predawn water potential. Models with the lowest dAIC within each species and variable were used in the analysis.

Model	AIC	dAIC	df	weight	Species
Polynomial	7,357.122	0.000000	4	0.933540390466734204544252407	*P. edulis*
Log	7,363.384	6.262607	3	0.040759170938215266710713536	*P. edulis*
Negative Exponential	7,364.307	7.184984	3	0.025700031162050402527841086	*P. edulis*
Linear	7,386.411	29.289237	3	0.000000407433000177014923488	*P. edulis*
Polynomial	7,556.594	0.000000	4	0.998812959778003994770756435	*J. monosperma*
Log	7,570.064	13.470260	3	0.001187010069195033228409142	*J. monosperma*
Negative Exponential	7,591.226	34.631601	3	0.000000030152800999361249316	*J. monosperma*
Linear	7,652.953	96.358766	3	0.000000000000000000001189718	*J. monosperma*

Supplementary Table 16. Results of the AIC test to find the best-fitting model when regressing % of maximum photosynthesis against predawn water potential. Although the best fitting model was a regression with an additive polynomial function, we could not determine the x-intercept from this model given the convex shape of the curve ($_{min}f(x) = x + x^2 > 0$). Any model with a dAIC < 7 is widely considered a valid model in the statistical literature and we therefore used the linear-logarithmic model (i.e. Log) in our analysis.

Species	Model	X-intercept	Variable
J. MONOSPERMA	Log	-6.861384	Growth
P. EDULIS	Log	-3.279419	Growth
J. monosperma	Negative Exponential	-2.974540	Growth
P. edulis	Negative Exponential	-3.937815	Growth
J. monosperma	Linear	-5.013598	Growth
P. edulis	Linear	-2.783840	Growth
P. edulis	Polynomial	-1.850000	Growth
J. monosperma	Polynomial	-3.250000	Growth
J. monosperma	Log	-6.755802	Photosynthesis
P. edulis	Log	-3.098974	Photosynthesis
J. monosperma	Negative Exponential	-1.761577	Photosynthesis
P. edulis	Negative Exponential	-4.104774	Photosynthesis
J. monosperma	Linear	-5.592805	Photosynthesis
P. edulis	Linear	-2.897830	Photosynthesis
P. edulis	Polynomial	-2.435000	Photosynthesis
J. monosperma	Polynomial	-4.815000	Photosynthesis

Supplementary Table 17. Effect of model choice on points of growth cessation and stomatal closure for both species. For all models (excepting polynomial), the x-intercept of the model was used to determine cessation points of photosynthesis and growth, respectively. Because the polynomial model has no x-intercept (it’s minimum > 0), we used the inflection point of the curve.

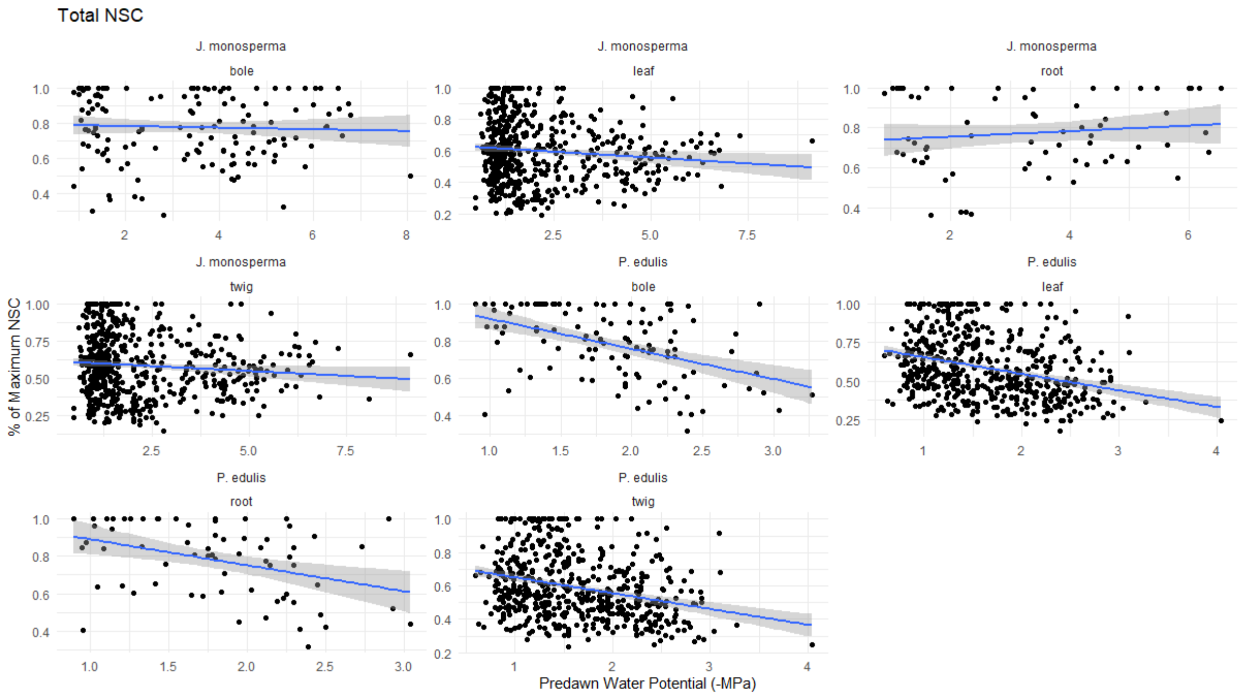

Supplementary Figure 8. Total NSC decreases in all tissues for *P. edulis*, for leaf only in *J. monosperma*. The results for *P. edulis* reflect the main results in this paper, suggesting total NSC decreases in *P. edulis*. Yet, for *J. monosperma*, there were tissue-specific responses. For instance, root NSC increases, suggesting osmotic adjustment of the roots allowing greater water uptake during drought. Leaf NSC decreased, highlighting the export of NSC to the roots. Grey areas around lines in indicate 95% confidence intervals.

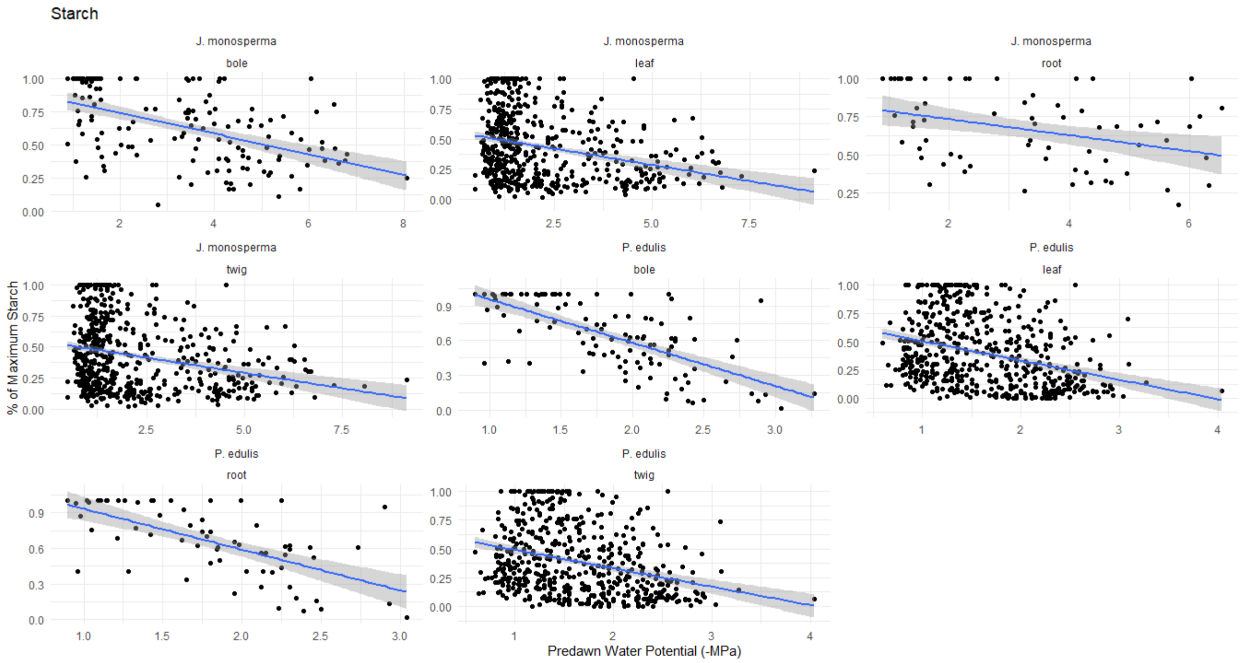

Supplementary Figure 9. Starch decreases in all tissues of both species, with increasing drought stress. For both species, the hydrolysis of starch for metabolic use in all tissues is highlighted. Grey areas around lines in indicate 95% confidence intervals.

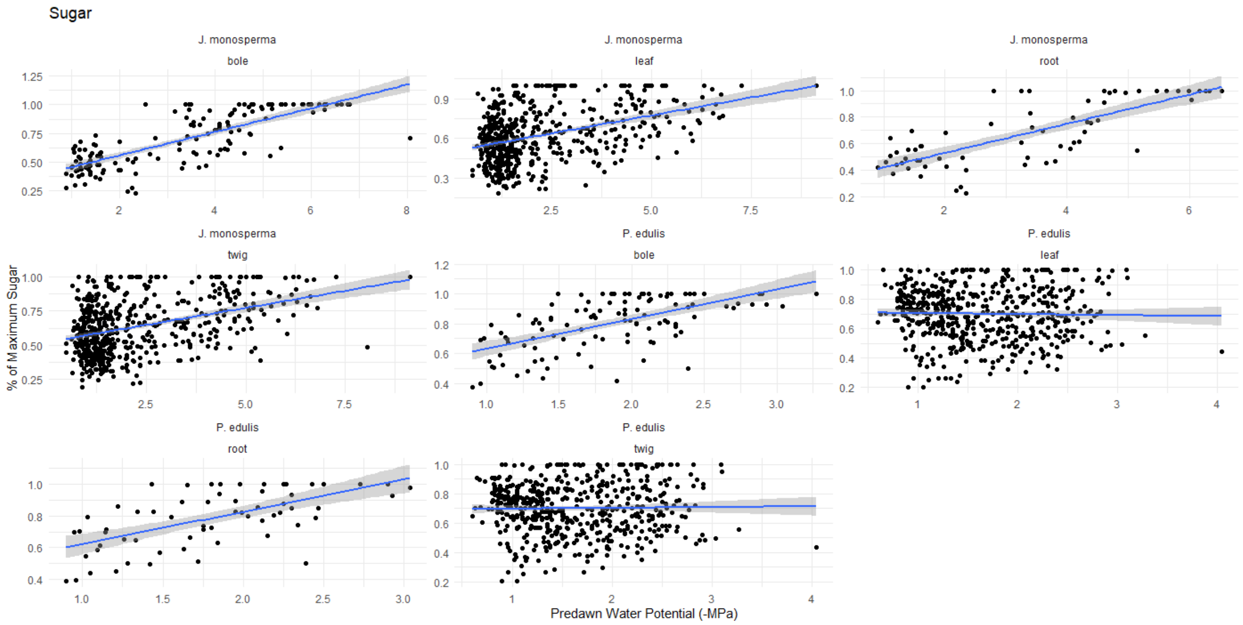

Supplementary Figure 10. Sugar concentrations increase with drought stress. For both species, sugar concentrations increased in all tissues (excluding *P. edulis* twig and leaf). Grey areas around lines in indicate 95% confidence intervals.

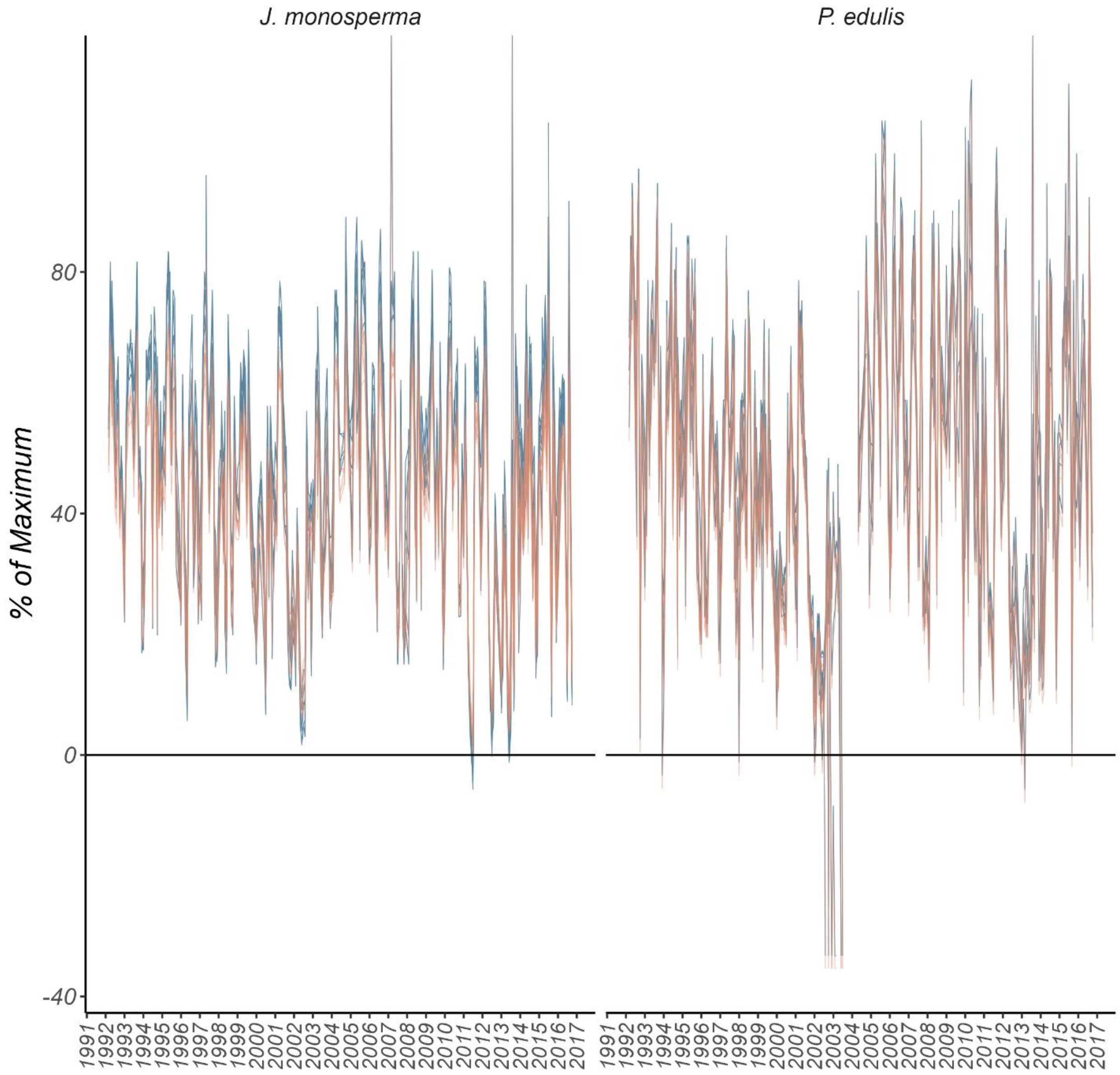

Supplementary Figure 11. Sink-source co-limitation may be rare under natural conditions. Data presented is extrapolated from monthly predawn water potential data at MDB, using the regression results in Fig. 1. Lines represent individual trees. Blue lines represent photosynthesis and red lines represent growth.

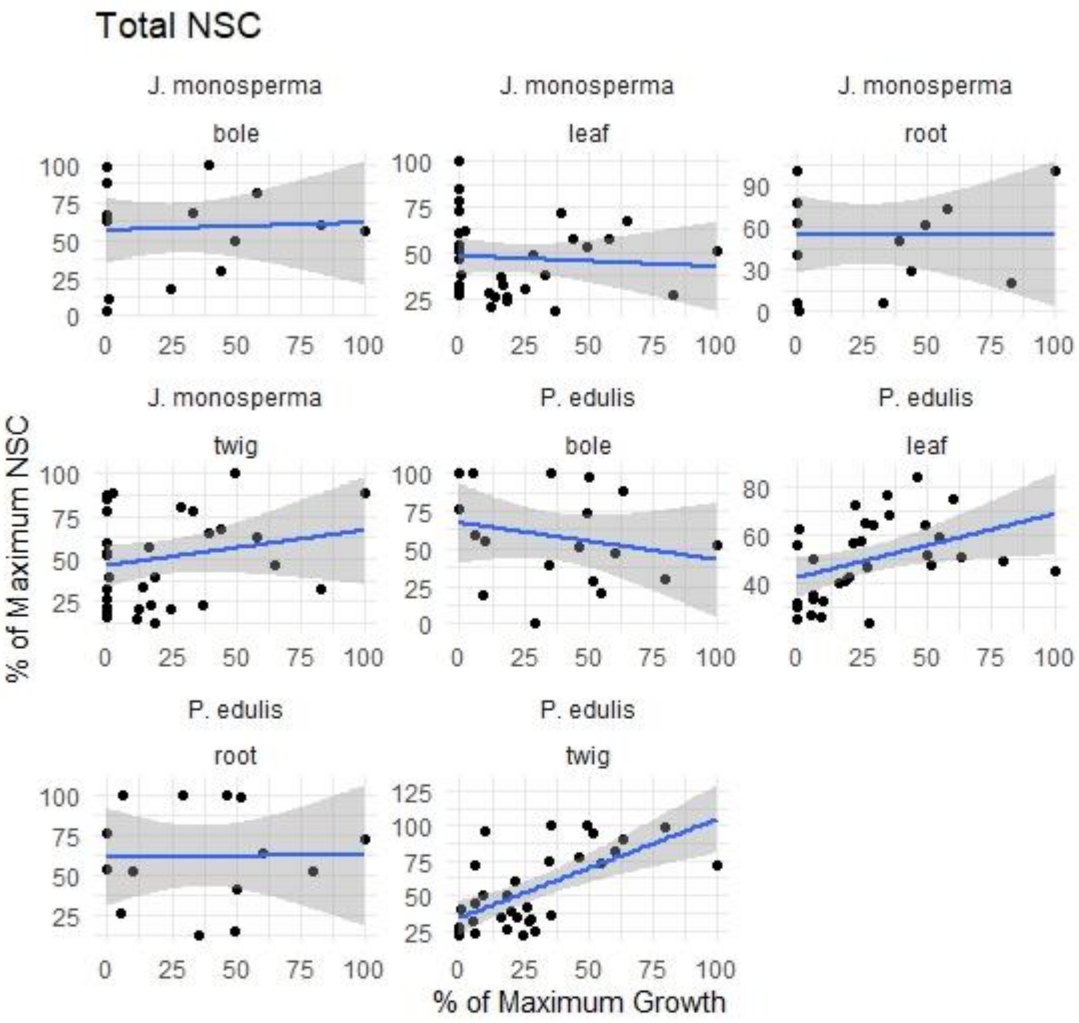

Supplementary Figure 12. Tissue-specific relationship between NSC and Growth. NSC had no significant relationship with growth in *J. monosperma* but declined only in *P. edulis* leaves and twigs as growth declined. Grey areas around lines in indicate 95% confidence intervals.

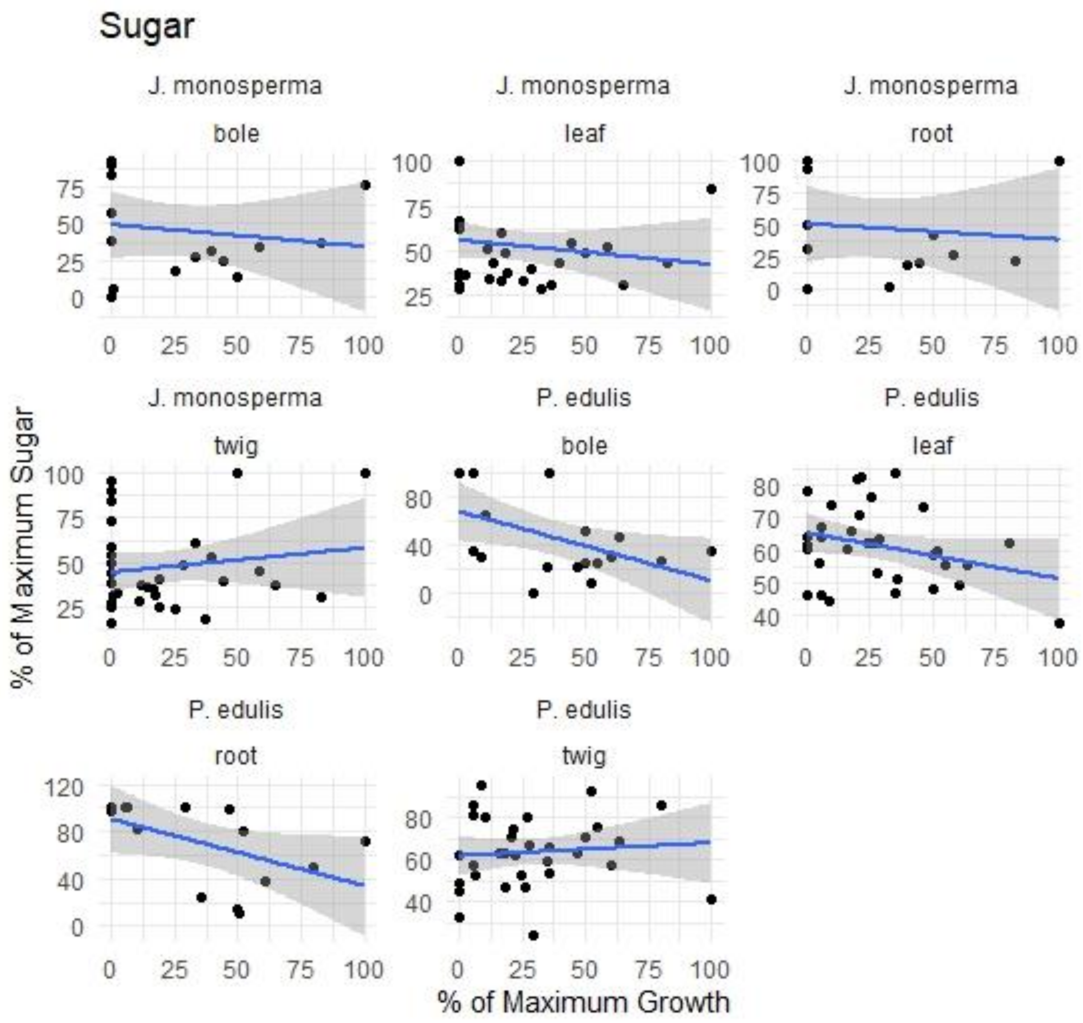

Supplementary Figure 13. Tissue-specific relationship between Sugar and Growth. Sugar had no significant relationship with growth in *J. monosperma* but increased only in the bole of *P. edulis* and not significantly in the roots of *P. edulis*. Grey areas around lines in indicate 95% confidence intervals.

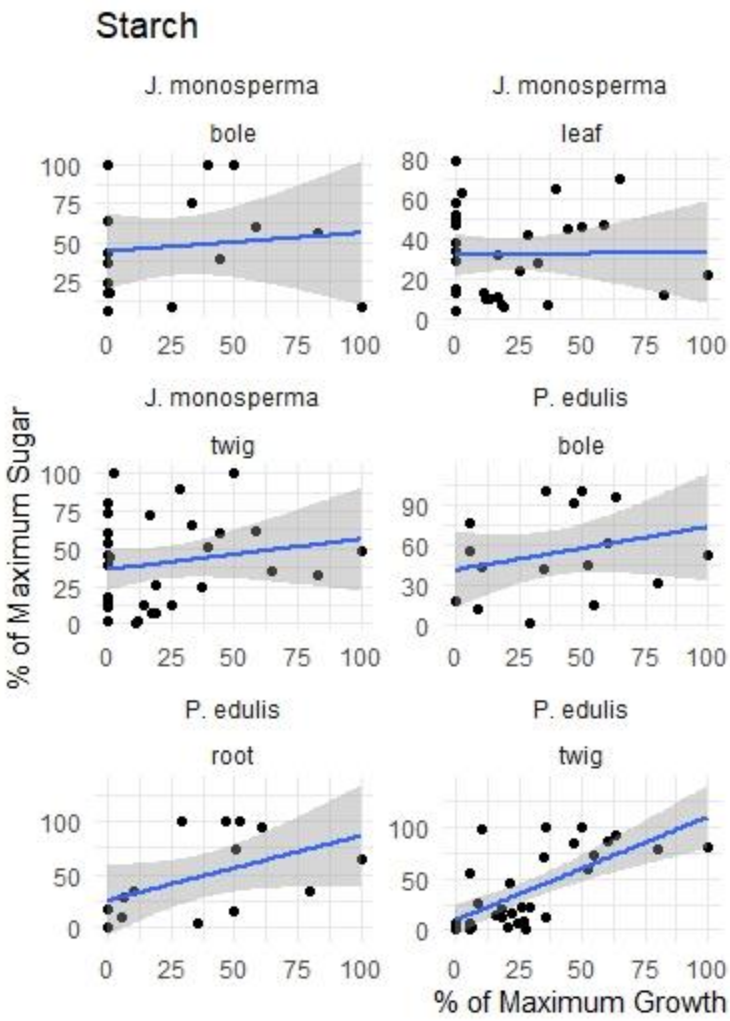

Supplementary Figure 14. Tissue-specific relationship between Starch and Growth. Starch had no significant relationship with growth in *J. monosperma* but decreased only in the leaves and twigs of *P. edulis*. Grey areas around lines in indicate 95% confidence intervals.
