# Supplementary material for: No carbon storage in growth-limited trees in a semi-arid woodland
Source: Nat Commun. 2023 Apr 7;14:1959. doi: 10.1038/s41467-023-37577-8 (PMC10081995; doi:10.1038/s41467-023-37577-8)
Supplement: Supplementary file 3 — Reporting Summary [file 41467_2023_37577_MOESM3_ESM.pdf]

nature portfolio

Corresponding author(s): R. Alex Thompson
Last updated by author(s): 3/9/2023

Reporting Summary

Nature Portfolio wishes to improve the reproducibility of the work that we publish. This form provides structure for consistency and transparency in reporting. For further information on Nature Portfolio policies, see our Editorial Policies and the Editorial Policy Checklist.

Statistics

For all statistical analyses, confirm that the following items are present in the figure legend, table legend, main text, or Methods section.

n/a	Confirmed	
☐	☒	The exact sample size (n) for each experimental group/condition, given as a discrete number and unit of measurement
☐	☒	A statement on whether measurements were taken from distinct samples or whether the same sample was measured repeatedly
☐	☒	The statistical test(s) used AND whether they are one- or two-sided<br>*Only common tests should be described solely by name; describe more complex techniques in the Methods section.*
☐	☒	A description of all covariates tested
☐	☒	A description of any assumptions or corrections, such as tests of normality and adjustment for multiple comparisons
☐	☒	A full description of the statistical parameters including central tendency (e.g. means) or other basic estimates (e.g. regression coefficient) AND variation (e.g. standard deviation) or associated estimates of uncertainty (e.g. confidence intervals)
☐	☒	For null hypothesis testing, the test statistic (e.g. F, t, r) with confidence intervals, effect sizes, degrees of freedom and P value noted<br>*Give P values as exact values whenever suitable.*
☒	☐	For Bayesian analysis, information on the choice of priors and Markov chain Monte Carlo settings
☒	☐	For hierarchical and complex designs, identification of the appropriate level for tests and full reporting of outcomes
☒	☐	Estimates of effect sizes (e.g. Cohen's d, Pearson's r), indicating how they were calculated

Our web collection on statistics for biologists contains articles on many of the points above.

Software and code

Policy information about availability of computer code

Data collection	No software was used
Data analysis	Data analysis was conducted in R (version 4.2.1) using the lm(), princomp(), and anova() functions. Data wrangling was conducting using a combination of base R and the dplyr package, while visualization was conducted using a combination of ggplot2, ggbiplot, and ggpubr packages. All analysis code can be found on github at: https://github.com/r-alex-thompson/SUMO.git

For manuscripts utilizing custom algorithms or software that are central to the research but not yet described in published literature, software must be made available to editors and reviewers. We strongly encourage code deposition in a community repository (e.g. GitHub). See the Nature Portfolio guidelines for submitting code & software for further information.

Data

Policy information about availability of data

All manuscripts must include a data availability statement. This statement should provide the following information, where applicable:
- Accession codes, unique identifiers, or web links for publicly available datasets
- A description of any restrictions on data availability
- For clinical datasets or third party data, please ensure that the statement adheres to our policy

All SUMO data can be accessed at the ESS Dive repository: https://data.ess-dive.lbl.gov/datasets/doi:10.15485/1440544

Human research participants

Policy information about studies involving human research participants and Sex and Gender in Research.

Reporting on sex and gender	*Use the terms sex (biological attribute) and gender (shaped by social and cultural circumstances) carefully in order to avoid confusing both terms. Indicate if findings apply to only one sex or gender; describe whether sex and gender were considered in study design whether sex and/or gender was determined based on self-reporting or assigned and methods used. Provide in the source data disaggregated sex and gender data where this information has been collected, and consent has been obtained for sharing of individual-level data; provide overall numbers in this Reporting Summary. Please state if this information has not been collected. Report sex- and gender-based analyses where performed, justify reasons for lack of sex- and gender-based analysis.*
Population characteristics	*Describe the covariate-relevant population characteristics of the human research participants (e.g. age, genotypic information, past and current diagnosis and treatment categories). If you filled out the behavioural & social sciences study design questions and have nothing to add here, write "See above."*
Recruitment	*Describe how participants were recruited. Outline any potential self-selection bias or other biases that may be present and how these are likely to impact results.*
Ethics oversight	*Identify the organization(s) that approved the study protocol.*

Note that full information on the approval of the study protocol must also be provided in the manuscript.

Field-specific reporting

Please select the one below that is the best fit for your research. If you are not sure, read the appropriate sections before making your selection.

☐ Life sciences ☐ Behavioural & social sciences ☒ Ecological, evolutionary & environmental sciences

For a reference copy of the document with all sections, see nature.com/documents/nr-reporting-summary-flat.pdf

Life sciences study design

All studies must disclose on these points even when the disclosure is negative.

Sample size	*Describe how sample size was determined, detailing any statistical methods used to predetermine sample size OR if no sample-size calculation was performed, describe how sample sizes were chosen and provide a rationale for why these sample sizes are sufficient.*
Data exclusions	*Describe any data exclusions. If no data were excluded from the analyses, state so OR if data were excluded, describe the exclusions and the rationale behind them, indicating whether exclusion criteria were pre-established.*
Replication	*Describe the measures taken to verify the reproducibility of the experimental findings. If all attempts at replication were successful, confirm this OR if there are any findings that were not replicated or cannot be reproduced, note this and describe why.*
Randomization	*Describe how samples/organisms/participants were allocated into experimental groups. If allocation was not random, describe how covariates were controlled OR if this is not relevant to your study, explain why.*
Blinding	*Describe whether the investigators were blinded to group allocation during data collection and/or analysis. If blinding was not possible, describe why OR explain why blinding was not relevant to your study.*

Behavioural & social sciences study design

All studies must disclose on these points even when the disclosure is negative.

Study description	*Briefly describe the study type including whether data are quantitative, qualitative, or mixed-methods (e.g. qualitative cross-sectional, quantitative experimental, mixed-methods case study).*
Research sample	*State the research sample (e.g. Harvard university undergraduates, villagers in rural India) and provide relevant demographic information (e.g. age, sex) and indicate whether the sample is representative. Provide a rationale for the study sample chosen. For studies involving existing datasets, please describe the dataset and source.*
Sampling strategy	*Describe the sampling procedure (e.g. random, snowball, stratified, convenience). Describe the statistical methods that were used to predetermine sample size OR if no sample-size calculation was performed, describe how sample sizes were chosen and provide a rationale for why these sample sizes are sufficient. For qualitative data, please indicate whether data saturation was considered, and what criteria were used to decide that no further sampling was needed.*

Data collection

Provide details about the data collection procedure, including the instruments or devices used to record the data (e.g. pen and paper, computer, eye tracker, video or audio equipment) whether anyone was present besides the participant(s) and the researcher, and whether the researcher was blind to experimental condition and/or the study hypothesis during data collection.

Timing

Indicate the start and stop dates of data collection. If there is a gap between collection periods, state the dates for each sample cohort.

Data exclusions

If no data were excluded from the analyses, state so OR if data were excluded, provide the exact number of exclusions and the rationale behind them, indicating whether exclusion criteria were pre-established.

Non-participation

State how many participants dropped out/declined participation and the reason(s) given OR provide response rate OR state that no participants dropped out/declined participation.

Randomization

If participants were not allocated into experimental groups, state so OR describe how participants were allocated to groups, and if allocation was not random, describe how covariates were controlled.

Ecological, evolutionary & environmental sciences study design

All studies must disclose on these points even when the disclosure is negative.

Study description

The Los Alamos Survival/Mortality (SUMO) experiment22,31–33 and MDB site20,34 have been described in detail previously by others. Briefly, the SUMO and MDB sites are located near Los Alamos, New Mexico USA, at elevations of 2150m and 2140m, respectively in piñon-juniper woodland just below the Pinus ponderosa forest ecotone. Pinus edulis and Juniperus monosperma dominate both sites, although scattered individuals of Quercus gambelli, P. ponderosa, J. deppeana and J. scopulorum can also be found at the SUMO site. A volcanic tuff parent material sits below the Hackroy clay loam soils that are found at both sites and can be found at depths ranging from 40-80 cm. The growing season occurs between April and October. Average 30-year temperature and precipitation were 10.1 C and 360mm, respectively. At both sites, trees growing naturally in the field (i.e., not planted but naturally recruited) were selected for observation and experimental study.

At SUMO, below-canopy precipitation removal structures and open-top heating chambers were installed during June 2012. A total of 64 individuals of P. edulis and J. monosperma (32 trees per species) growing in the ground were selected and placed into one of five treatments (5-7 trees per species in each), however due to a lack of growth data, only four treatments are considered in this paper. The ambient treatment consisted of trees exposed to ambient temperature and precipitation. The heat treatment was implemented by placing open top chambers around selected trees to create an average increase of 4.8°C above ambient temperatures. Drought trees were exposed to ambient temperatures within a precipitation removal structure that diverted ~45% of precipitation away from these trees. Heat + Drought trees were exposed to both the 4.8°C temperature increase and the precipitation removal. Continuous measurement of site climatic conditions using two weather stations, in addition to within-chamber measurements, allowed control of chamber conditions using heating and air-conditioning units. In general, all measurements were made on the same trees such that comparisons of growth, photosynthesis, water potential, or NSC are robust. However, every parameter was not measured on every tree. Where all measurements in an analysis are not present for every tree in the study, those trees are excluded from that analysis. All trees assess in this study at SUMO had measurements of growth, photosynthesis, water potential, and NSC.

At the MDB site, five trees each of P. edulis and J. monosperma were selected for long term monitoring in March 1992, and two additional P. edulis trees were added in 1994. In 2003, all seven measured P. edulis trees died from drought and bark beetle attack, and five surviving replacements were selected in 2004. Measurements from one tree were switched to another in 2008. Several J. monosperma were added to measurements in subsequent years: five in 2007, and three in 2015.

Research sample

12 Juniperus monosperma, 13 Pinus edulis. All trees were reproductively mature and ranged in size from 0.5 m to 5.5m tall and crown widths that ranged from 1-5 m. Trees included in the study were selected based on two criteria: large enough to support several physiological measurements for multiple years, and no less than 10m from the nearest edge of drought structures.

Sampling strategy

Mesocosm experiments on mature trees are rare because of difficulties in exposing such large organisms to experimental conditions. Our experiment simulated drought and heat conditions in the field, which is inherently limited by species density (e.g., we could not move trees into our plots). In this case, sample size was not a limiting factor (n>10) for the statistical tests we used (primarily linear regression, F-tests, and Analysis of Variance, given the number of factor levels in our models (2).

Data collection

Radial Growth Measurements and Calculations

At SUMO, tree radial growth was measured as outlined by Manrique-Alba et al. 33. Briefly, from May through September in 2013 and 2014, a linear variable displacement transducer (LVDT) was attached to the upper bole of 11 individuals of J. monosperma and 12 individuals of P. edulis using a rectangular frame that was attached directly to the tree using screws. Trees were selected from Heat, Heat + Drought, Drought, and Ambient treatments. Dead bark was gently removed from the site where the sensor contacted the tree, though a thin layer was left as to protect the phloem and prevent water loss.

Data from the LVDT is a relative metric of change in diameter over time. Upon installation, each LVDT was set to “zero” the night of the first day of the measurement period before any growth occurred. This established the reference point from which measured growth would deviate. Because diurnal fluxes of stem diameter make calculating growth difficult, we considered growth initiation to have occurred when the maximum stem diameter exceeded that of the previous day’s maximum, for each tree. When maximum stem diameter did not exceed the previous day’s maximum, we considered growth to have stopped. No direct measurements of growth were made on trees at the MDB site.

Water potential and Photosynthesis

At SUMO, xylem water potential and foliar gas exchange were determined for 11 individuals of J. monosperma and 12 individuals of P. edulis (2011 - 2017). Two twig samples were collected every three months from each tree before sunrise and the xylem water tension was measured using a Scholander pressure chamber (PMS Instruments, Albany, OR, USA). The level of water stress for each tree was quantified as the average predawn water potential (ψ_pd) of both stems. At MDB, xylem water potential was measured

every month between 1992 – 2016. Measurements were made on 5-6 individuals of J. monosperma from 1992 – 2012, and 11-14 individuals of J. monosperma from 2013 – 2016. Between 1992 – 2016, water potential measurements were made on 5-7 individuals of P. edulis.

At SUMO only, net photosynthesis and gas exchange were measured on the south-facing, sun-exposed side of each tree using a Li-Cor LI-6400 (Lincoln, NE, USA). Needles from each tree were measured under chamber conditions set of 380ppm CO2, 1500 mol m^(-2) s^(-1) light-saturating photosynthetic photon flux density (PPFD), temperatures between 20-25°C, and 0% relative humidity. These conditions closely matched those of the outside environment, where temperatures ranged from 13 – 30°C and 750 – 1800 mmol m^(-2) s^(-1) PPFD. After two minutes of steady-state gas exchange, measurements were recorded, and needle samples were collected to determine leaf area. Gas exchange data was corrected using leaf area measurements made on a Li-Cor LI3100C area meter. No gas exchange measurements were made on the trees at MDB.

Non-Structural Carbohydrates

Beginning on March 14th, 2012, and ending on October 13th, 2016 at SUMO, leaf and stem (twig; phloem and bark) tissue samples were collected for each tree four times each year to capture seasonal changes associated with spring dormancy break, mid-summer drought, monsoon wet-season, and post-monsoon dry-down. Stem samples were from recent growth, dated from 0 to 5 years old for P. edulis. Bole and root samples (also including bark) were collected using an increment borer once per year during the dry season, in June. All samples were collected between 11:30 and 13:00, mitigating any influence of diurnal variation in NSC on our measurements (Gersony et al. 2020, Tixier et al. 2018). Upon collection, samples were placed into liquid nitrogen and transported to the lab in dry ice. Samples were kept stored at -70°C until analysis, when they were microwaved for 5 min at 800W and placed in a drying oven for 48h at 65°C. All samples were ground using a ball mill and woody tissues were preground using a Wiley Mini-Mill. To assay NSC, we used the protocol outlined by Dickman et al. 35, as developed from the methods of Hoch et al. 36. This method has been verified to produce reasonably accurate and precise measurements of NSC, defined as glucose, fructose, sucrose, and starch.37 ~12mg of finely ground sample was placed into a deep-well plate with 1.6 mL deionized water and placed into a 100C water bath for 1h. An NAD-linked enzymatic assay was used in combination with spectral assessment at 340 nm for NSC quantification. To analyze NSCs at the whole-tree or canopy scales we averaged NSC concentrations from each respective tissue. For example, to calculate canopy NSC, sugar, and starch, we averaged the NSC from stem and needle tissues. A similar approach was used to estimate whole-tree NSC for June. No NSCs were measured on the trees at MDB.

HDA, DDB, ADC, LTD, CG, AMA, AMT, and NGM collected the data.

Timing and spatial scale	All treatments were initiated on 11 June 2012. Growth was measured daily, beginnin May 1st and ending Sept 30th 2013, and from May 1st to Sept 8th 2014. NSC was measured monthly every year from March 14 2012 to Oct 13 2016. Water potential was measured monthly from March 10 2011 - May 9 2017. Photosynthesis was measured monthly from April 4 2012 - Mar 21 2017. This relatively frequent sampling for the duration of the experiment allowed us to capture any seasonal effect on these parameters as well as fully characterize the physiological response of these trees to experimental treatments. All samples were collected at the scale of individual trees.
Data exclusions	Data was excluded from this manuscript only if it was determined to be an outlier in the analysis. Outlier criteria was set using 3x Cook's distance, as described in our manuscript. The rational for this was to remove any overwhelming effect on regression coefficients on the main results. this is a standard statistical procedure to remove data points that appear to be erroneous.
Reproducibility	Due to the high cost of the experiment and relatively long duration, no attempts to reproduce this experiment have occurred. However, many similar drought experiments have since occurred both in this ecosystem (i.e., Sevilleta National Wildlife Refuge) and in the tropics.
Randomization	Plots were not randomly placed since construction constraints dictated where specific treatments could be installed. Within plots, ttrees that fit the size criteria outlined above were randomly selected and placed into their respective treatments.
Blinding	Blinding was not relevant to this study since it is inherently observational.

Did the study involve field work? ☒ Yes ☐ No

Field work, collection and transport

Field conditions	The Los Alamos Survival/Mortality (SUMO) experiment22,31–33 and MDB site20,34 have been described in detail previously by others. Briefly, the SUMO and MDB sites are located near Los Alamos, New Mexico USA, at elevations of 2150m and 2140m, respectively in piñon-juniper woodland just below the Pinus ponderosa forest ecotone. Pinus edulis and Juniperus monosperma dominate both sites, although scattered individuals of Quercus gambelli, P. ponderosa, J. deppeana and J. scopulorum can also be found at the SUMO site. A volcanic tuff parent material sits below the Hackroy clay loam soils that are found at both sites and can be found at depths ranging from 40-80 cm. The growing season occurs between April and October. Average 30-year temperature and precipitation were 10.1 C and 360mm, respectively. At both sites, trees growing naturally in the field (i.e., not planted but naturally recruited) were selected for observation and experimental study.<br>At SUMO, below-canopy precipitation removal structures and open-top heating chambers were installed during June 2012. A total of 64 individuals of P. edulis and J. monosperma (32 trees per species) growing in the ground were selected and placed into one of five treatments (5-7 trees per species in each), however due to a lack of growth data, only four treatments are considered in this paper. The ambient treatment consisted of trees exposed to ambient temperature and precipitation. The heat treatment was implemented by placing open top chambers around selected trees to create an average increase of 4.8°C above ambient temperatures. Drought trees were exposed to ambient temperatures within a precipitation removal structure that diverted ~45% of precipitation away from these trees. Heat + Drought trees were exposed to both the 4.8°C temperature increase and the precipitation removal. Continuous measurement of site climatic conditions using two weather stations, in addition to within-chamber measurements, allowed control of chamber conditions using heating and air-conditioning units. In general, all measurements were made on the same trees such that

comparisons of growth, photosynthesis, water potential, or NSC are robust. However, every parameter was not measured on every tree. Where all measurements in an analysis are not present for every tree in the study, those trees are excluded from that analysis. All trees assess in this study at SUMO had measurements of growth, photosynthesis, water potential, and NSC.

At the MDB site, five trees each of P. edulis and J. monosperma were selected for long term monitoring in March 1992, and two additional P. edulis trees were added in 1994. In 2003, all seven measured P. edulis trees died from drought and bark beetle attack, and five surviving replacements were selected in 2004. Measurements from one tree were switched to another in 2008. Several J. monosperma were added to measurements in subsequent years: five in 2007, and three in 2015.

Location	Los Alamos, New Mexico, USA (elev. 2140 - 2150m)
Access & import/export	All samples were collected with explicit permission from Los Alamos National Laboratory and the US Department of Energy. Site access at SUMO is highly restricted, and permits allowing the experiment and access to the field site were awarded via DOE contract #: AC52-06NA25396.
Disturbance	This research significantly disturbed the ecosystem in which it was performed. This disturbance was necessary, as the primary aim of this experiment was to understand the response of this ecosystem to experimental disturbance (here, heat and drought).

Reporting for specific materials, systems and methods

We require information from authors about some types of materials, experimental systems and methods used in many studies. Here, indicate whether each material, system or method listed is relevant to your study. If you are not sure if a list item applies to your research, read the appropriate section before selecting a response.

Materials & experimental systems

n/a	Involved in the study
☒	☐ Antibodies
☒	☐ Eukaryotic cell lines
☒	☐ Palaeontology and archaeology
☒	☐ Animals and other organisms
☒	☐ Clinical data
☒	☐ Dual use research of concern

Methods

n/a	Involved in the study
☒	☐ ChIP-seq
☒	☐ Flow cytometry
☒	☐ MRI-based neuroimaging

Antibodies

Antibodies used	*Describe all antibodies used in the study; as applicable, provide supplier name, catalog number, clone name, and lot number.*
Validation	*Describe the validation of each primary antibody for the species and application, noting any validation statements on the manufacturer's website, relevant citations, antibody profiles in online databases, or data provided in the manuscript.*

Eukaryotic cell lines

Policy information about cell lines and Sex and Gender in Research

Cell line source(s)	*State the source of each cell line used and the sex of all primary cell lines and cells derived from human participants or vertebrate models.*
Authentication	*Describe the authentication procedures for each cell line used OR declare that none of the cell lines used were authenticated.*
Mycoplasma contamination	*Confirm that all cell lines tested negative for mycoplasma contamination OR describe the results of the testing for mycoplasma contamination OR declare that the cell lines were not tested for mycoplasma contamination.*
Commonly misidentified lines (See ICLAC register)	*Name any commonly misidentified cell lines used in the study and provide a rationale for their use.*

Palaeontology and Archaeology

Specimen provenance	*Provide provenance information for specimens and describe permits that were obtained for the work (including the name of the issuing authority, the date of issue, and any identifying information). Permits should encompass collection and, where applicable, export.*
Specimen deposition	*Indicate where the specimens have been deposited to permit free access by other researchers.*
Dating methods	*If new dates are provided, describe how they were obtained (e.g. collection, storage, sample pretreatment and measurement), where they were obtained (i.e. lab name), the calibration program and the protocol for quality assurance OR state that no new dates are*

provided.

☐ Tick this box to confirm that the raw and calibrated dates are available in the paper or in Supplementary Information.

Ethics oversight	*Identify the organization(s) that approved or provided guidance on the study protocol, OR state that no ethical approval or guidance was required and explain why not.*

Note that full information on the approval of the study protocol must also be provided in the manuscript.

Animals and other research organisms

Policy information about studies involving animals; ARRIVE guidelines recommended for reporting animal research, and Sex and Gender in Research

Laboratory animals	*For laboratory animals, report species, strain and age OR state that the study did not involve laboratory animals.*
Wild animals	*Provide details on animals observed in or captured in the field; report species and age where possible. Describe how animals were caught and transported and what happened to captive animals after the study (if killed, explain why and describe method; if released, say where and when) OR state that the study did not involve wild animals.*
Reporting on sex	*Indicate if findings apply to only one sex; describe whether sex was considered in study design, methods used for assigning sex. Provide data disaggregated for sex where this information has been collected in the source data as appropriate; provide overall numbers in this Reporting Summary. Please state if this information has not been collected. Report sex-based analyses where performed, justify reasons for lack of sex-based analysis.*
Field-collected samples	*For laboratory work with field-collected samples, describe all relevant parameters such as housing, maintenance, temperature, photoperiod and end-of-experiment protocol OR state that the study did not involve samples collected from the field.*
Ethics oversight	*Identify the organization(s) that approved or provided guidance on the study protocol, OR state that no ethical approval or guidance was required and explain why not.*

Note that full information on the approval of the study protocol must also be provided in the manuscript.

Clinical data

Policy information about clinical studies

All manuscripts should comply with the ICMJEguidelines for publication of clinical research and a completedCONSORT checklist must be included with all submissions.

Clinical trial registration	*Provide the trial registration number from ClinicalTrials.gov or an equivalent agency.*
Study protocol	*Note where the full trial protocol can be accessed OR if not available, explain why.*
Data collection	*Describe the settings and locales of data collection, noting the time periods of recruitment and data collection.*
Outcomes	*Describe how you pre-defined primary and secondary outcome measures and how you assessed these measures.*

Dual use research of concern

Policy information about dual use research of concern

Hazards

Could the accidental, deliberate or reckless misuse of agents or technologies generated in the work, or the application of information presented in the manuscript, pose a threat to:

No	Yes	
☒	☐	Public health
☒	☐	National security
☒	☐	Crops and/or livestock
☒	☐	Ecosystems
☒	☐	Any other significant area

Experiments of concern

Does the work involve any of these experiments of concern:

No	Yes	
☒	☐	Demonstrate how to render a vaccine ineffective
☒	☐	Confer resistance to therapeutically useful antibiotics or antiviral agents
☒	☐	Enhance the virulence of a pathogen or render a nonpathogen virulent
☒	☐	Increase transmissibility of a pathogen
☒	☐	Alter the host range of a pathogen
☒	☐	Enable evasion of diagnostic/detection modalities
☒	☐	Enable the weaponization of a biological agent or toxin
☒	☐	Any other potentially harmful combination of experiments and agents

ChIP-seq

Data deposition

☐ Confirm that both raw and final processed data have been deposited in a public database such as GEO.

☐ Confirm that you have deposited or provided access to graph files (e.g. BED files) for the called peaks.

Data access links<br>*May remain private before publication.*	*For "Initial submission" or "Revised version" documents, provide reviewer access links. For your "Final submission" document, provide a link to the deposited data.*
Files in database submission	*Provide a list of all files available in the database submission.*
Genome browser session<br>(e.g. UCSC)	*Provide a link to an anonymized genome browser session for "Initial submission" and "Revised version" documents only, to enable peer review. Write "no longer applicable" for "Final submission" documents.*

Methodology

Replicates	*Describe the experimental replicates, specifying number, type and replicate agreement.*
Sequencing depth	*Describe the sequencing depth for each experiment, providing the total number of reads, uniquely mapped reads, length of reads and whether they were paired- or single-end.*
Antibodies	*Describe the antibodies used for the ChIP-seq experiments; as applicable, provide supplier name, catalog number, clone name, and lot number.*
Peak calling parameters	*Specify the command line program and parameters used for read mapping and peak calling, including the ChIP, control and index files used.*
Data quality	*Describe the methods used to ensure data quality in full detail, including how many peaks are at FDR 5% and above 5-fold enrichment.*
Software	*Describe the software used to collect and analyze the ChIP-seq data. For custom code that has been deposited into a community repository, provide accession details.*

Flow Cytometry

Plots

Confirm that:

☐ The axis labels state the marker and fluorochrome used (e.g. CD4-FITC).

☐ The axis scales are clearly visible. Include numbers along axes only for bottom left plot of group (a 'group' is an analysis of identical markers).

☐ All plots are contour plots with outliers or pseudocolor plots.

☐ A numerical value for number of cells or percentage (with statistics) is provided.

Methodology

Sample preparation	*Describe the sample preparation, detailing the biological source of the cells and any tissue processing steps used.*
Instrument	*Identify the instrument used for data collection, specifying make and model number.*

Software	*Describe the software used to collect and analyze the flow cytometry data. For custom code that has been deposited into a community repository, provide accession details.*
Cell population abundance	*Describe the abundance of the relevant cell populations within post-sort fractions, providing details on the purity of the samples and how it was determined.*
Gating strategy	*Describe the gating strategy used for all relevant experiments, specifying the preliminary FSC/SSC gates of the starting cell population, indicating where boundaries between "positive" and "negative" staining cell populations are defined.*

☐ Tick this box to confirm that a figure exemplifying the gating strategy is provided in the Supplementary Information.

Magnetic resonance imaging

Experimental design

Design type	*Indicate task or resting state; event-related or block design.*
Design specifications	*Specify the number of blocks, trials or experimental units per session and/or subject, and specify the length of each trial or block (if trials are blocked) and interval between trials.*
Behavioral performance measures	*State number and/or type of variables recorded (e.g. correct button press, response time) and what statistics were used to establish that the subjects were performing the task as expected (e.g. mean, range, and/or standard deviation across subjects).*

Acquisition

Imaging type(s)	*Specify: functional, structural, diffusion, perfusion.*
Field strength	*Specify in Tesla*
Sequence & imaging parameters	*Specify the pulse sequence type (gradient echo, spin echo, etc.), imaging type (EPI, spiral, etc.), field of view, matrix size, slice thickness, orientation and TE/TR/flip angle.*
Area of acquisition	*State whether a whole brain scan was used OR define the area of acquisition, describing how the region was determined.*

Diffusion MRI ☐ Used ☐ Not used

Preprocessing

Preprocessing software	*Provide detail on software version and revision number and on specific parameters (model/functions, brain extraction, segmentation, smoothing kernel size, etc.).*
Normalization	*If data were normalized/standardized, describe the approach(es): specify linear or non-linear and define image types used for transformation OR indicate that data were not normalized and explain rationale for lack of normalization.*
Normalization template	*Describe the template used for normalization/transformation, specifying subject space or group standardized space (e.g. original Talairach, MNI305, ICBM152) OR indicate that the data were not normalized.*
Noise and artifact removal	*Describe your procedure(s) for artifact and structured noise removal, specifying motion parameters, tissue signals and physiological signals (heart rate, respiration).*
Volume censoring	*Define your software and/or method and criteria for volume censoring, and state the extent of such censoring.*

Statistical modeling & inference

Model type and settings	*Specify type (mass univariate, multivariate, RSA, predictive, etc.) and describe essential details of the model at the first and second levels (e.g. fixed, random or mixed effects; drift or auto-correlation).*
Effect(s) tested	*Define precise effect in terms of the task or stimulus conditions instead of psychological concepts and indicate whether ANOVA or factorial designs were used.*

Specify type of analysis: ☐ Whole brain ☐ ROI-based ☐ Both

Statistic type for inference (See Eklund et al. 2016)	*Specify voxel-wise or cluster-wise and report all relevant parameters for cluster-wise methods.*
Correction	*Describe the type of correction and how it is obtained for multiple comparisons (e.g. FWE, FDR, permutation or Monte Carlo).*

Models & analysis

n/a	Involved in the study
☒	☐ Functional and/or effective connectivity
☒	☐ Graph analysis
☒	☐ Multivariate modeling or predictive analysis

Functional and/or effective connectivity	*Report the measures of dependence used and the model details (e.g. Pearson correlation, partial correlation, mutual information).*
Graph analysis	*Report the dependent variable and connectivity measure, specifying weighted graph or binarized graph, subject- or group-level, and the global and/or node summaries used (e.g. clustering coefficient, efficiency, etc.).*
Multivariate modeling and predictive analysis	*Specify independent variables, features extraction and dimension reduction, model, training and evaluation metrics.*
